# Dramatic and Elusive Resonant Lattice Kerker Effect in the Nonlinear Response of Plasmonic Lattices

**DOI:** 10.1002/nap2.70157

**Published:** 2026-06-11

**Authors:** Sergejs Boroviks, Benjamin S. Garcia, Ebru Buhara, Olivier J. F. Martin

**Affiliations:** ^1^ Nanophotonics and Metrology Laboratory Swiss Federal Institute of Technology Lausanne (EPFL) Lausanne Switzerland

**Keywords:** generalized Kerker effect, plasmonic metasurface, second‐harmonic generation, surface lattice resonance

## Abstract

This article reports an experimental study of a plasmonic metasurface that supports the resonant lattice Kerker effect in the near‐infrared wavelength range, manifesting as the suppression of reflection in a narrow spectral range. Such directional scattering is explained by the interaction of electric dipole and quadrupole modes that are simultaneously excited in the meta‐atoms and also gives rise to a substantial near‐field enhancement. Although in theory this enhancement is anticipated to drastically amplify second‐harmonic generation, we show that experimentally this effect is rather elusive due to its extreme sensitivity to experimental conditions. Our experimental results are supported by numerical simulations, and physical insights into the observed effects are provided using a multipole decomposition analysis. This work indicates experimental pitfalls that must be taken into account in the design of nonlinear plasmonic metasurfaces, and suggests how to circumvent them.

## Graphical abstract

Plasmonic metasurface that supports the resonant lattice Kerker effect, manifesting as the suppression of reflection in a narrow spectral range due to the simultaneous excitation of electric dipole and quadrupole modes, also gives rise to a substantial near‐field enhancement. In theory, this enhancement leads to a dramatic amplification of second‐harmonic generation; however, experimental characterization reveals the elusiveness of the effect.

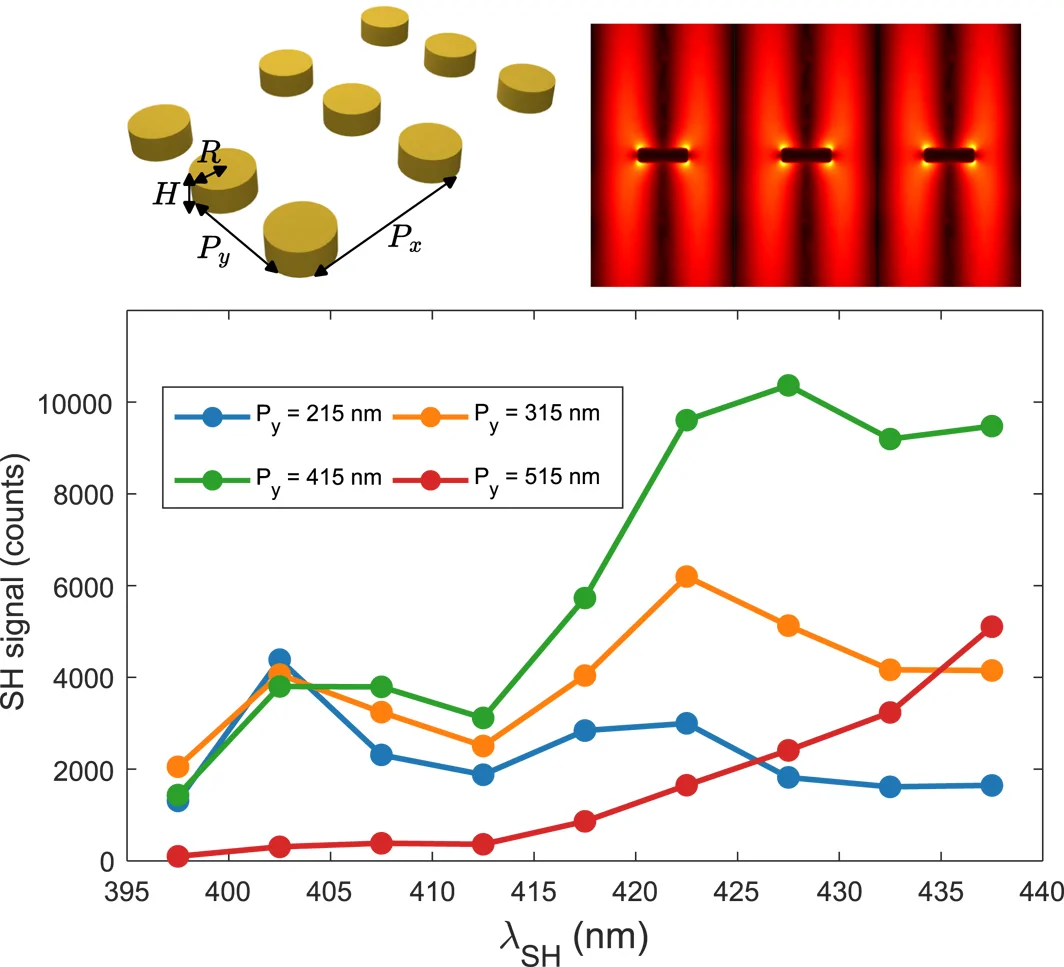

## 1 Introduction

Photonic metasurfaces—two‐dimensional arrays of optical nanostructures, so‐called meta‐atoms—are becoming a ubiquitous tool for light manipulation at the nanoscale in both linear [1, 2, 3] and nonlinear [4, 5, 6] regimes. Among the large variety of materials used in metasurface designs, noble metals are widespread because they support surface plasmon resonances, which allow to achieve large near‐field enhancement in extremely small volumes [7, 8]. Although this near‐field enhancement comes at the price of Ohmic losses, various strategies exist to mitigate or, on the contrary, to exploit them [9, 10].

Despite having a subwavelength thickness, plasmonic metasurfaces can feature multiple functionalities simultaneously, which is not attainable with conventional optical components [11, 12]. For example, metasurfaces were shown to perform nonlinear light generation simultaneously with beam stirring [13, 14], focusing [15, 16], holography [17, 18], as well as to explore chiral [19, 20] and anisotropic [21] properties. We invite the interested readers to consult recent comprehensive reviews on the topic [22, 23].

In many cases, the aforementioned functionalities of plasmonic metasurfaces are achieved by making use of either the resonances of individual meta‐atoms or the collective resonances of the entire array. One of such collective resonances, known as surface lattice resonance (SLR) [24, 25, 26], is often used to achieve sharp features in scattering spectra [27, 28, 29, 30]. A key advantage of SLRs is their relatively high Q‐factor, with reported experimental values up to 2340 [31]. This is rooted in the fact that a SLR is the result of the far‐field interference of the meta‐atoms’ dipolar resonances, which is a Fano‐type interference phenomenon, yielding a sharp, asymmetric spectral line shape [32, 33, 34].

It was shown that SLR can significantly enhance nonlinear optical processes [35, 36, 37, 38, 39], in particular second‐harmonic generation (SHG). SHG is a second‐order nonlinear process that (within the dipolar approximation) occurs only in noncentrosymmetric crystals. However, even in centrosymmetric materials, such as noble metals, centrosymmetry is broken at the surface, giving rise to a surface‐SHG. In plasmonic systems, a substantial surface‐SHG can be obtained due to large enhancement of the normal component of the electric field at the metal nanoparticle surface [40, 41, 42, 43], this effect can also be described in a homogenized picture, using effective susceptibilities [44, 45].

Intrinsically, dipolar resonances feature low absorption, which theoretically cannot exceed 50% of the incident power [46]; however, for some applications high absorption is desirable. This can be achieved by making use of the magnetic and electric higher‐order multipolar moments (quadrupole, octopole etc.) [47, 48]. Furthermore, engineering the far‐field interference of the higher‐order multipoles provides a versatile control over the scattering directionality through the generalized Kerker effect [49, 50]. When it is realized in a periodic metasurface, it is also often referred to as the resonant lattice Kerker effect [51] or metasurface analog of electromagnetically induced transparency [52]. A number of recent articles showed, in theory, a complete suppression of reflection in a plasmonic metasurface leveraging this effect [53, 54, 55, 56]; however, an experimental demonstration, to the best of our knowledge, is still missing.

In this manuscript, we experimentally and numerically study a plasmonic metasurface that exhibits a resonant lattice Kerker effect at the fundamental wavelength and its implications for SHG. Although it was theoretically predicted that at the Kerker condition the field enhancement associated with the simultaneous excitation of several multipoles in a plasmonic metasurface may amplify SHG in a very narrow spectral range [57], our findings indicate that this condition is challenging to achieve experimentally. The observation of the effect requires a coherent excitation of the electric dipole and quadrupole modes, which gives rise to directional light scattering in the far‐field and strong near‐field enhancement in the linear regime. However, it appears to be extremely sensitive to the excitation conditions and fabrication imperfections. Additionally, in the considered system, the resonant lattice Kerker effect is also accompanied with a strong absorption, which may surpass the benefits of the strong near‐field enhancement, as follows from general considerations on SHG and the trade‐off between light absorption and near‐field enhancement at the surface [58, 59].

## 2 Results and Discussion

We consider a metasurface that comprises gold cylinders with radius R=95nm and height H=60nm, arranged in a rectangular lattice with periodicity $P_{x}$ and $P_{y}$ embedded in a homogeneous glass background, as illustrated in Figure 1a. A homogeneous background is used to avoid additional complications related to the reflections at the interfaces between different dielectrics, which were shown to alter the lattice resonances [53, 60]. First, we study the linear optical response of the system using full‐wave simulations (see the Methods section for details). In simulations, the permittivity of the cylinders is described using interpolated experimental data [61], whereas the infinite background medium has the refractive index $n_{b}=1.49$ (in the experiment the substrate and superstate thicknesses are much larger than the considered wavelengths (see Methods section), and hence, the assumption of an infinite uniform background is justified).

**FIGURE 1:**
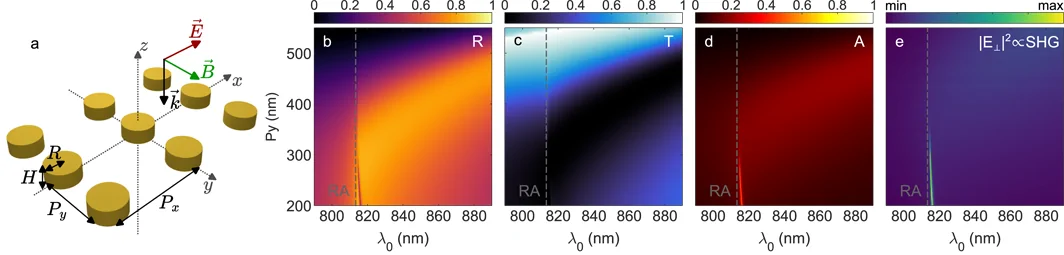
(a) Schematic diagram of the considered metasurface with indicated dimensions. (b–e) Pseudo‐color plots as a function of wavelength $\lambda_{0}$ and periodicity $P_{y}$ for zeroth‐order reflectance (b), transmittance (c), absorptance (d) and square of the normal component of the electric field integrated over the nanoparticle surface (e). Dashed lines in panels (b–e) indicate the spectral position of the RA.

Figure 1b–e shows pseudo‐color plots of the simulated 0th diffraction order reflection, R, transmission, T, and absorption, A as well as the square of the intensity of the normal component of the electric field, $|E_{⊥}{|}^{2}$, integrated over the cylinder surface, for a metasurface with $P_{x}=545\;nm$ and $P_{y}$ varying in the 200–550 nm range, illuminated with a x‐polarized plane wave at normal incidence.

Although the transmission spectrum (Figure 1c) does not exhibit any sharp features, the reflectance (Figure 1b) shows a sharp dip at a free space wavelength $\lambda_{0}\approx 815\;nm$, which is also accompanied with a strong absorption (Figure 1d). The absorption peak also corresponds with a strong enhancement of the normal component of the electric field at the nanoparticle surface (Figure 1e), which determines the induced nonlinear surface polarization:

$$P_{⊥}^{s}\left(r,2\omega \right)=\varepsilon_{0}\delta \left(r-r_{s}\right)\chi_{⊥⊥⊥}^{\left(2\right)}E_{⊥}\left(r,\omega \right)E_{⊥}\left(r,\omega \right),$$

where $\varepsilon_{0}$ is the vacuum permittivity, δ a delta function, r the position vector, $r_{s}$ the position vector of the surface and $\chi_{⊥⊥⊥}^{\left(2\right)}$ is the normal component of the surface second‐order susceptibility. Apart from $\chi_{⊥⊥⊥}^{\left(2\right)}$, other components of the second‐order susceptibility may exist (including higher‐order multipole bulk contributions); however, in this work, we consider only $\chi_{⊥⊥⊥}^{\left(2\right)}$ because it was shown to be the strongest, by orders of magnitude, for most plasmonic systems [41, 62, 63, 64]. The intensity of the SHG is proportional to the square of the nonlinear polarization, $I_{\text{SHG}}\propto |P_{⊥}^{s}\left(r,2\omega \right){|}^{2}$ [65, 66] and therefore, a strong amplification of SHG is expected at the position of the resonance.

As will be shown next, the strong and abrupt resonance visible in Figure 1b,d is due to the resonant lattice Kerker effect [50, 51, 53, 55, 56, 67]. It is important to note that the spectral position of the resonant lattice Kerker effects is nearly independent of $P_{y}$, although its visibility decreases with increasing periodicity. In plasmonic metasurfaces, this effect occurs in the vicinity of the Rayleigh anomaly (RA), which corresponds to the onset of diffraction $\left(\lambda_{\text{RA}}=n_{b}P_{x}=812.05\;nm\right)$, but always at a longer wavelength [68]. This implies that the Kerker effect occurs in a nondiffractive regime. Importantly, as can be inferred from Supporting Information S1: Figure S2c, there are no additional resonances at the second‐harmonic wavelength that could affect the nonlinear optical response of the system: the linear reflection spectra are almost flat except for appearance of the second‐order RA below approx 406 nm.

With the intention of getting additional insights into the physical origin of the sharp dip in reflection observed in Figure 1b, we perform a multipole decomposition analysis [69, 70]. Following the method introduced by Savinov et al. [71], a metasurface can be represented as an array of interacting multipolar scatterers. As can be seen in Figure 2a, the metasurface exhibits a broad electric dipole (ED) resonance, whose amplitude is modulated by the $P_{y}$ periodicity. This resonance is associated with the fundamental localized surface plasmon resonance of an individual nanoparticle. Additionally, at around $\lambda_{0}\approx 815\;nm$, a spectrally narrow electric quadrupole (EQ) is excited, whose amplitude is also modulated by the same parameter $P_{y}$, as visible in Figure 2b. This resonance is associated with a spatially extended (also called nonlocal) mode. We have also computed the magnetic dipole and quadrupole contributions; however, they appear to be negligible. Therefore, the spectrally narrow suppression of the reflection is caused by the simultaneous excitation of the ED and EQ multipoles, which results in a localization and large enhancement of the electric field in the vicinity of the metasurface (see the computed electric near‐field distribution in Figure 2d that corresponds to the maximum electric quadrupole contribution). In turn, the electric dipole resonance also exhibits an appreciable near‐field enhancement (corresponding to $\lambda_{0}=844\;nm$ and $P_{y}=415\;nm$, plotted in Figure 2c), which is approximately 1.85 times smaller than the enhancement at the dipole resonance. As can be seen from the component‐wise multipole decomposition in Supporting Information S1: Figure S3, the ED is along x‐axis, and EQ is oriented in the xz‐plane, that is, ${ED}_{x}$ and ${EQ}_{xz}$ components are dominant. Given that ${ED}_{x}$ and ${EQ}_{xz}$ have opposite phase in the +z‐direction (Figure 2e,f), they interfere destructively in the far‐field, resulting in the suppressed scattering in the backward direction, as illustrated in Figure 2g, is actually known as the generalized Kerker effect [49, 50]. Importantly, in the absence of electric quadrupole, the broad electric dipole resonance also shows a substantial electric near‐field enhancement (Figure 2c), albeit somewhat modest in comparison with that at the Kerker effect (Figure 2d); however, this simple dipolar resonance has lower losses and, as will be discussed further, turns out to be more robust to the various experimental imperfections.

**FIGURE 2:**
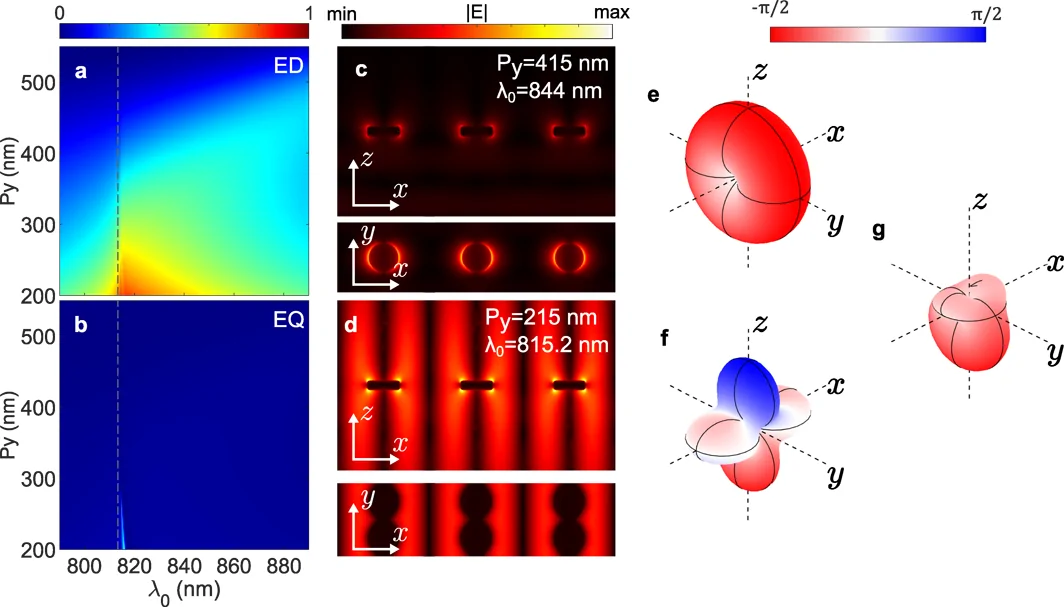
Multipole analysis of the metasurface response. (a) Electric dipole, (b) electric quadrupole. Dashed lines in (a) and (b) indicate the spectral position of the RA. (c, d) Near‐field amplitude distribution in the vicinity of the metasurface at the dipole resonance in the xz and yz planes (c, $\lambda_{0}=844\;nm$ and $P_{y}=415\;nm$) and at the generalized Kerker effect condition (d, $\lambda_{0}=815.2\;nm$ and $P_{y}=215\;nm$). (e, f) 3D distribution of the normalized scattered electric field (the distance from the origin indicates the amplitude while pseudo‐colors represent the phase) of ED (e) and EQ (f), whereas (g) shows the sum of the ED and EQ contributions, leading to the scattering suppression in the backward direction (i.e., generalized Kerker effect).

In order to study these effects experimentally, we fabricated a set of metasurface samples with the same parameters as in the simulations (R=95nm and H=60nm) and $P_{y}$ periodicity varying from 215 to 515 nm across the different samples, using electron beam lithography and ion‐beam etching of the predeposited gold thin film. This method is proven to be advantageous as compared with the commonly employed lift‐off technique [72]. Optical micrographs of the fabricated samples are presented in Supporting Information S1: Figure S1 and details on fabrication procedures are outlined in the Methods section.

Figure 3 shows the experimental characterization of the fabricated samples (an exemplary scanning electron microscope (SEM) image is shown in Figure 3a). The linear reflectance (Figure 3b) is in fairly good qualitative agreement with the simulation results (Figure 1b): the spectra exhibit a dip in reflection at approx. 815 nm, whose visibility is tuned by the metasurface periodicity along the y‐axis. As in the simulations, the strongest reduction is achieved with $P_{y}=215\;nm$; however, the experimental spectra feature a much wider dip. There are multiple reasons for the observed resonance broadening. First, it can be attributed to fabrication imperfections that cause a distribution of the nanoparticle sizes and thus reduce the quality factor of the quadrupole resonance that is very sensitive to the geometric dimensions. Besides, the metasurface sample has a finite size (100×100 periods which corresponds to approx. 50×50μm), whereas in the simulation infinite periodicity is considered. Furthermore, in the linear reflection spectroscopy we use a standard halogen lamp, which has a low (on the order of 1 μm) coherence length and a finite size of the excitation spot (approx. 20 μm) that even further reduces the number of metasurface periods interacting with the incident light. It is known that lattice resonances, associated with the spatially extended modes, are particularly sensitive to all these factors [31, 73, 74, 75], which makes challenging the experimental retrieval of the theoretically predicted narrow linewidth. In contrast, the excitation of the ED, associated with the individual, localized surface plasmon resonance, is less sensitive to all the aforementioned factors.

**FIGURE 3:**
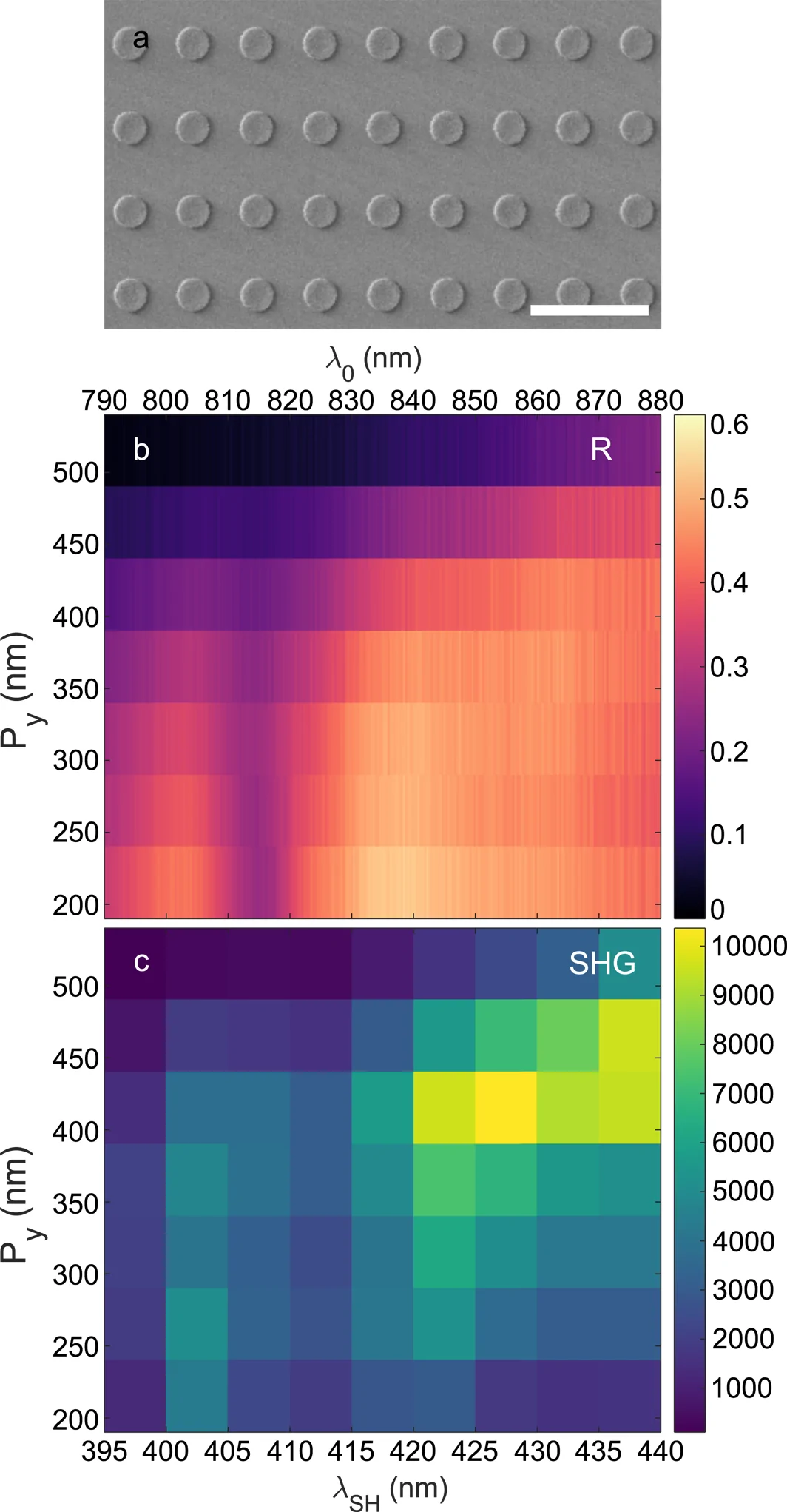
Experimental characterization of the fabricated samples. (a) SEM image of a metasurface sample with $P_{x}=545\;nm$ and $P_{y}=515\;nm$ (scale bar: 1 μm). (b) Measured linear reflectance spectra and (c) SHG response as a function of wavelength $\lambda_{0}$ and periodicity $P_{y}$ (with fixed $P_{x}=545\;nm$).

Next, we investigate the SHG response of our metasurface samples (the raw measured spectra are provided in Supporting Information S1: Figure S5 and the experimental procedures are detailed in the Methods section). To check the nonlinearity of the measured signal, we have performed excitation power dependence measurements (Supporting Information S1: Figure S6) and observed nearly quadratic dependence (slope of 1.94), which is expected for second‐order nonlinear processes. In contrast with the good agreement observed in the linear regime, the experimental SHG spectral map (Figure 3c) exhibits a drastically different behavior from the simulated $|E_{⊥}{|}^{2}$ enhancement spectra shown in Figure 1e. In our simulation (as well as previous works by Han et al. [57]), an order of magnitude SHG enhancement is expected at the lattice Kerker effect conditions because of a large near‐field enhancement associated with the excitation of the quadrupole mode (see Figure 2). However, experimentally we observe a suppression of the SHG signal for all the periods at $\lambda_{\text{SH}}\approx 413\;nm$. On the other hand, the sample with $P_{y}=415\;nm$ exhibits a maximum at $\lambda_{\text{SH}}\approx 425\;nm$, which is in partial agreement with the simulation results (note the broad and weak, but noticeable, increase of $|E_{⊥}{|}^{2}$ at longer wavelengths in Figure 1e).

We attribute this discrepancy between the experiment and the simulations to several factors. First, as mentioned above, a finite size of the metasurface sample and the excitation spot, as well as small deviations from the design dimensions and dispersion of the nanoparticle sizes, weaken the excitation of the spectrally sharp electric quadrupole resonance. SHG is known to be extremely sensitive to such perturbations [76, 77, 78, 79]. Additionally, we underscore the role of the resonant absorption due to the excitation of the electric quadrupole mode. Because of the known trade‐off between resonant absorption and SHG [58, 59], the spectrally sharp resonant absorption at the Kerker effect takes over the benefits associated with the enhancement of the electric field at the surface. Lastly, but most importantly, we note that the SHG at the resonant lattice Kerker effect condition is extremely sensitive to the angle of incidence of the excitation light. This is shown in Figure 4, which compares simulated SHG spectra for $P_{y}=215,315,415$ and 515 nm samples at normal and 0.5° angles of incidence, along with the experimental data. At normal incidence, the metasurface with $P_{y}=215\;nm$ exhibits a very sharp SHG peak just next to the RA wavelength (fundamental wavelength $\lambda_{0}=812\;nm$, corresponding to second‐harmonic wavelength $\lambda_{\text{SH}}=406\;nm$). As the angle of incidence is tilted just by a tiny 0.5◦, this sharp feature diminishes by two orders of magnitude, leaving behind only a small blue‐shifted peak. In fact, such strong dependence on the angle of incidence is expected for the nonlocal metasurfaces that support spatially extended modes [80, 81, 82]. In contrast, the electric dipole resonance yields a broad, yet distinct peak in SHG, which is especially pronounced for the structure with $P_{y}=415\;nm$ and is nearly independent of the angle of incidence. Besides, this resonance is also accompanied with a relatively strong absorption in the linear regime (Figure 1d).

**FIGURE 4:**
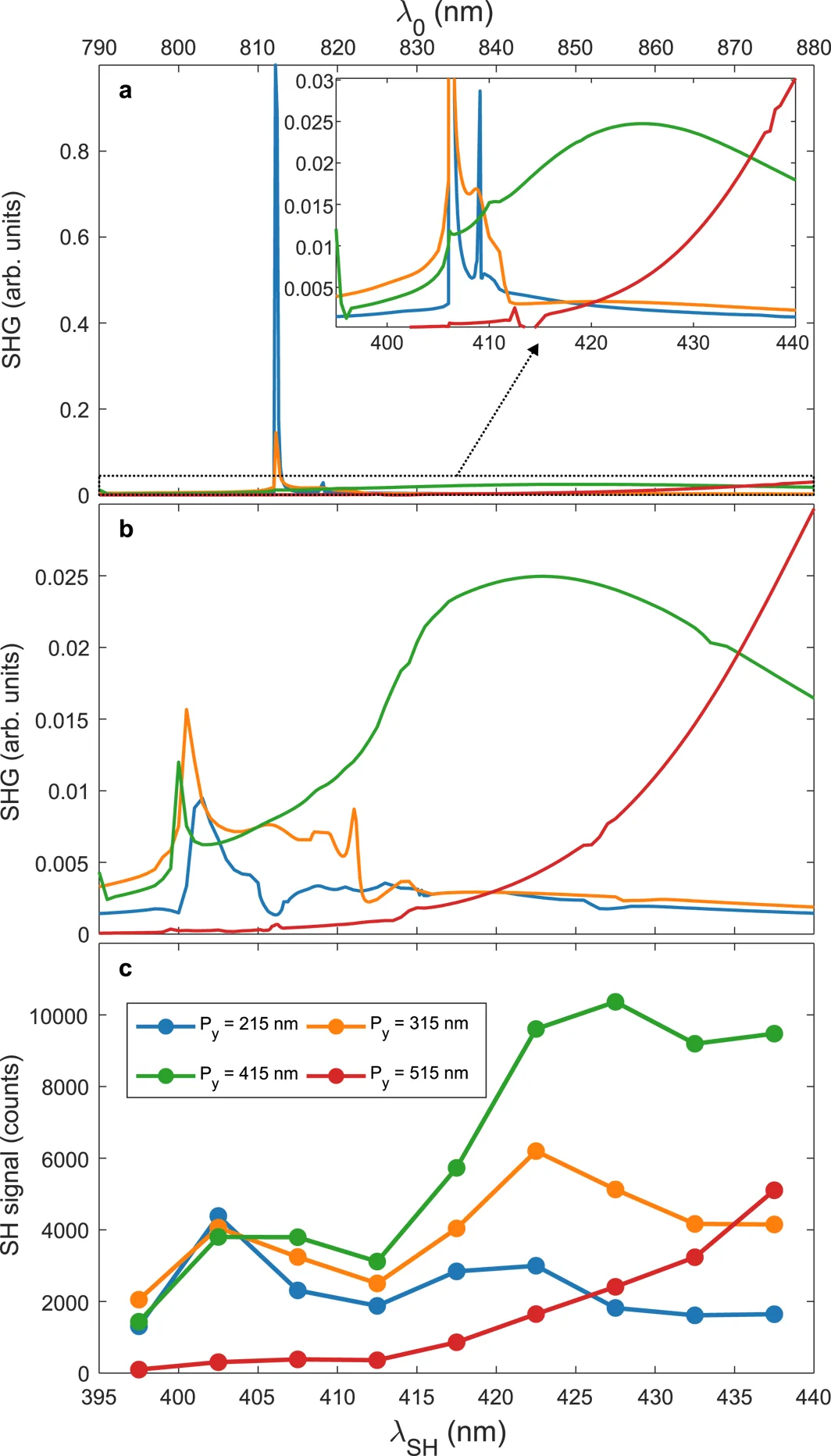
Analysis of SHG at normal and slightly oblique incidences. (a, b) Simulated SHG response for a metasurface with $P_{y}=215,315,415$ and 515 nm, and $P_{x}=545\;nm$ at (a) normal and (b) 0.5° angles of incidence; the inset in a shows a zoom‐in of the same spectrum to highlight features that are not visible on a large scale. (c) Experimentally obtained SHG spectra for the metasurfaces with the same geometric parameters as in (a) and (b). The same color coding is used consistently across all subfigures to indicate metasurface samples with different $P_{y}$ periodicities.

Clearly, experimental SHG spectra (Figure 4c) are in a better qualitative agreement with the simulations for the slightly tilted angle of incidence (Figure 4b). This is because in a microscopic experiment, the excitation beam is rather a quasi‐plane‐wave and characterized by a distribution of propagation angles, which effectively smears‐out the strongly dispersive and spectrally sharp effect. In our setup, the sample is illuminated with a gaussian beam that has numerical aperture (NA) 0.018, which corresponds to approximately a 0.5◦ half‐angle, as shown in the Fourier‐plane characterization of the beam in Supporting Information S1: Figure S7. Because the NA of the objective is large (0.9), whereas angular spread of the incident beam is relatively small, it is challenging to position it exactly in the center of the objective's pupil. In our experiment, we tried to perform centering by maximizing the nonlinear signal intensity, which is extremely sensitive to minute misalignment. The uncertainty due to this misalignment is nontrivial to estimate quantitatively, although we suppose that it is at least on the order of FWHM of the beam's angular size, as indicated in Supporting Information S1: Figure S7e. Due to the trade‐off between the excitation power, the excitation spot size at the sample and at the back‐focal (Fourier) plane, it is practically unfeasible to achieve strictly normal illumination in such a microscope experiment. Indeed, a tighter focusing of the beam in the objective back‐focal plane increases the size of the excitation spot, thus requiring more power, which may damage the objective. On the other hand, reducing the beam size in the back‐focal plane with a pinhole substantially limits the excitation power. These constraints limit the applicability of the resonant lattice Kerker effect for SHG enhancement in plasmonic metasurfaces in experimental conditions. On the contrary, the less dramatic enhancement offered by the dipole resonance offers a more robust way to enhance SHG, as it is less sensitive to the angle of incidence and fabrication imperfections. Additionally, it is important to note that SHG light exhibits diffraction (Supporting Information S1: Figure S7d), since the effective SH wavelength in glass, $\lambda_{\text{SH}}/n$ is smaller than both periodicities $P_{x}$ and $P_{y}$.

## 3 Conclusion

In this work, we experimentally and theoretically investigated the linear and nonlinear optical responses of plasmonic metasurfaces that exhibit a generalized Kerker effect. Although from theoretical predictions, the lattice Kerker effect at the fundamental wavelength is expected to enhance the SHG in a very narrow spectral range, our results indicate that this enhancement is very difficult to achieve in practice due to the strong sensitivity of the Kerker effect to the angle of incidence of the excitation light due to its nonlocal nature, as well as the critical role of absorption. In contrast, electric dipole resonances, which do not exhibit such strong sensitivity to the experimental imperfections and feature a lower absorption together with a substantial near‐field enhancement, turn out to be a more robust mechanism for resonant SHG in plasmonic metasurafaces.

## 4 Methods

### 4.1 Numerical Simulations

Electromagnetic simulations were performed in frequency domain using the wave optics module of the commercially available finite element numerical solver Comsol (version 6.2). Linear and nonlinear simulations are performed in consecutive steps that are coupled through the Comsol Multiphysics interface. First, the linear response is obtained in a standard manner: a plane‐wave launched from an excitation port in a three‐dimensional simulation domain bounded by periodic boundary conditions on the sides and perfectly matched layers on top and bottom. The transmitted wave is captured by the receiver port, which allows us to calculate reflection, transmission, and absorption spectra. Furthermore the surface‐normal components of the electric field just inside the metal domain (i.e., just below the gold‐dielectric interfaces) is evaluated to obtain the sources for the nonlinear simulations. Then, the SHG simulation at the doubled frequency is performed following the method proposed by Reddy et al. [83]. In short, because the surface polarization is not supported by Comsol, it is transformed into surface currents at the metal surface that act as sources in the second simulation step. Then, the radiated SHG power is obtained by integrating the normal component of the Poynting vector at a 2 μm distance from the origin of the simulation domain where the center of the particle is located.

### 4.2 Sample Fabrication

We employed the ion beam etching (IBE) technique which has been shown to yield high‐quality nanostructures with sharp features [72]. The substrate used was a float glass wafer (Guinchard Switzerland) pre‐coated with 2 nm Ti (for adhesion purpose), 60 nm‐thick Au and 20 nm Cr films using an e‐beam assisted evaporator (Alliance‐Concept EVA 760). The metasurface patterns are exposed in a negative tone e‐beam resist (HSQ, XR‐1541‐006 from DuPont), using electron beam lithography (Raith EBPG5000+). Subsequently, the exposed patterns are transferred from the resist to the gold using a low‐power argon IBE (Veeco Nexus IBE350, operated at a 300 V acceleration voltage). Importantly, the IBE is operated in a pulsed mode: 10 s of etching followed by 30 s of cooling to avoid damaging the sample by substrate overheating. The typical overall IBE process time is 60 s, and the etching depth is controlled in situ using a mass‐spectrometer, which allows real‐time monitoring of the etched material composition: the etching process is stopped as soon as the Au flux drops to a minimum. Finally, the sample is coated with 1 μm of ${\text{SiO}}_{2}$ using radio‐frequency sputtering (Pfeiffer SPIDER 600), and a cover slip glass is attached to it using a drop of immersion oil (Olympus IMMOIL‐F30CC) to create an optically homogeneous background.

### 4.3 Linear Optical Characterization

The microscopic reflection spectroscopy measurements of the samples were performed using an inverted microscope (Olympus IX73, using a × 100, NA = 0.9 objective) and a spectrograph (Andor SR‐303i‐A, equipped with Newton 971 EMCCD camera). The setup has a Fourier (i.e., scattering angle‐resolved) imaging capability and thus allows us to select and record the spectrum only of the light reflected in a direction close to normal. Supporting Information S1: Figure S2 shows comparison of the measured and simulated reflection spectra for the sample with the same periodicities $P_{y}$ as those presented in Figure 4.

### 4.4 Nonlinear Optical Characterization

The nonlinear characterization of the fabricated metasurface samples was performed using a custom‐built nonlinear microscopy setup, schematics are shown in Supporting Information S1: Figure S4. The laser used for the excitation was a Mira 900 by Coherent, producing ∼120 fs pulses with tunable central wavelength. The horizontally polarized (i.e., x‐polarized in the metasurface frame) laser beam was focused onto the back‐focal plane of a ×100, NA=0.9 apochromatic objective (Leica HCX PL APO 767016), thus creating a quasi‐plane‐wave like illumination with approximately 5 μm full width at half maximum (FWHM) diameter of the excitation spot. In the Fourier plane, the excitation beam has a FWHM size of NA = 0.018, which corresponds to a half‐angle of 0.5◦. The characterization of the excitation beam is presented in Supporting Information S1: Figure S7. The same objective was used for reflected nonlinear light collection, which, after passing through a dichroic mirror and a short‐pass filter, was recorded with a spectrometer (Andor SR‐303i, equipped with a charge‐coupled device camera). Additionally, we placed a slit in the Fourier plane of the nonlinear image to ensure collection of only the 0 and ±1 diffraction orders along the x‐axis, because for samples with small periodicities $P_{x}$ higher diffraction orders were also collected by the objective (see Supporting Information S1: Figure S7d). As the excitation wavelength was tuned with steps of 10 nm in the 795–875 nm range, the spectrum of the nonlinear signal was recorded in a broad 350–630 nm range. This allows us to ensure during post‐processing that the recorded signal comes solely from the SHG, filtering out nonlinear photoluminescence. The data presented in Figure 3b was obtained by integrating the SHG spectrum in a ± 2 nm range of the corresponding $\lambda_{\text{SH}}$. The raw nonlinear spectra are presented in Supporting Information S1: Figure S5.

## Author Contributions

**Sergejs Boroviks:** writing – review and editing, writing – original draft, conceptualization, investigation, methodology, data curation, supervision, formal analysis, validation, visualization. **Benjamin S. Garcia:** conceptualization, methodology, data curation, investigation, visualization, validation, formal analysis, writing – review and editing. **Ebru Buhara:** investigation, validation, writing – review and editing. **Olivier J. F. Martin:** conceptualization, writing – review and editing, project administration, funding acquisition, supervision, methodology.

## Conflicts of Interest

The authors declare no conflicts of interest.

## Supporting information


Supporting Information S1

## Data Availability

The data that support the findings of this study are available from the corresponding author upon reasonable request.
