# Supplementary material for: Dramatic and Elusive Resonant Lattice Kerker Effect in the Nonlinear Response of Plasmonic Lattices
Source: Nanophotonics. 2026 Jun 11;15(12):e70157. doi: 10.1002/nap2.70157 (PMC13642115; doi:10.1002/nap2.70157)
Supplement: Supplementary file 1 [file NAP2-15-e70157-s001.pdf]

Supporting Information for:
Dramatic and elusive resonant lattice Kerker effect in the nonlinear response of plasmonic lattices

Sergejs Boroviks*, Benjamin S. Garcia, Ebru Buhara, and Olivier J. F. Martin*
Nanophotonics and Metrology Laboratory,
*Swiss Federal Institute of Technology Lausanne (EPFL), Lausanne, Switzerland**

(Dated: May 15, 2026)

CONTENTS

S1. Optical microscope images of the fabricated samples 2

S2. Linear reflection spectra 3

S3. Component-wise multipole decomposition 4

S4. Nonlinear microscopy setup 5

S5. Power and spectral dependence of the nonlinear signal 6

S6. Characterization of the excitation and SHG spot 8

* sergejs.boroviks@epfl.ch; olivier.martin@epfl.ch

S1. OPTICAL MICROSCOPE IMAGES OF THE FABRICATED SAMPLES

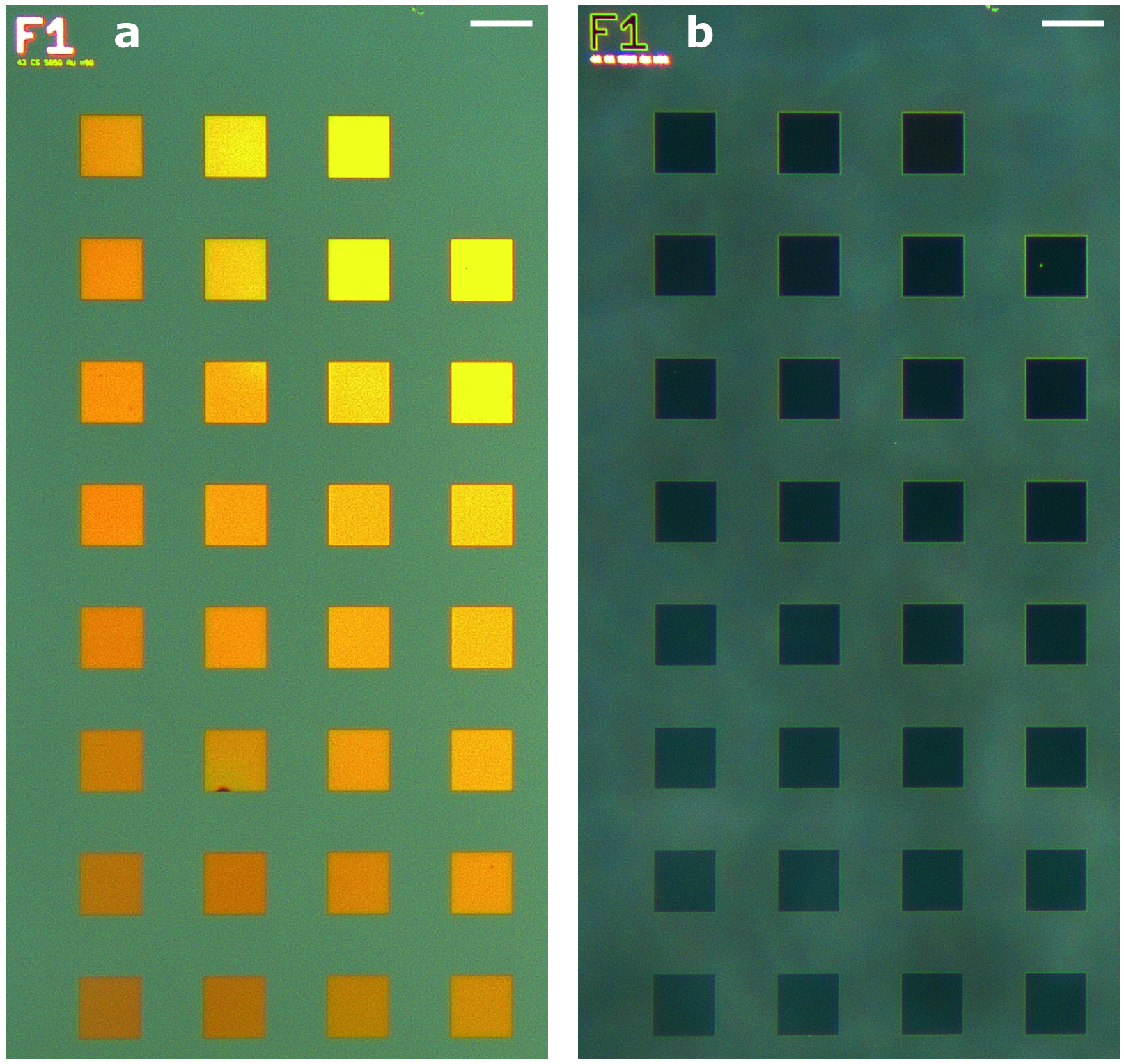

FIG. S1. **a** Bright-field and **b** dark-field images of the fabricated samples; scale bars: 50 µm. The columns correspond to different nominal nanocylinder radii R, whereas the rows correspond to different values of P_x, nominally from 215 to 515 nm.

S2. LINEAR REFLECTION SPECTRA

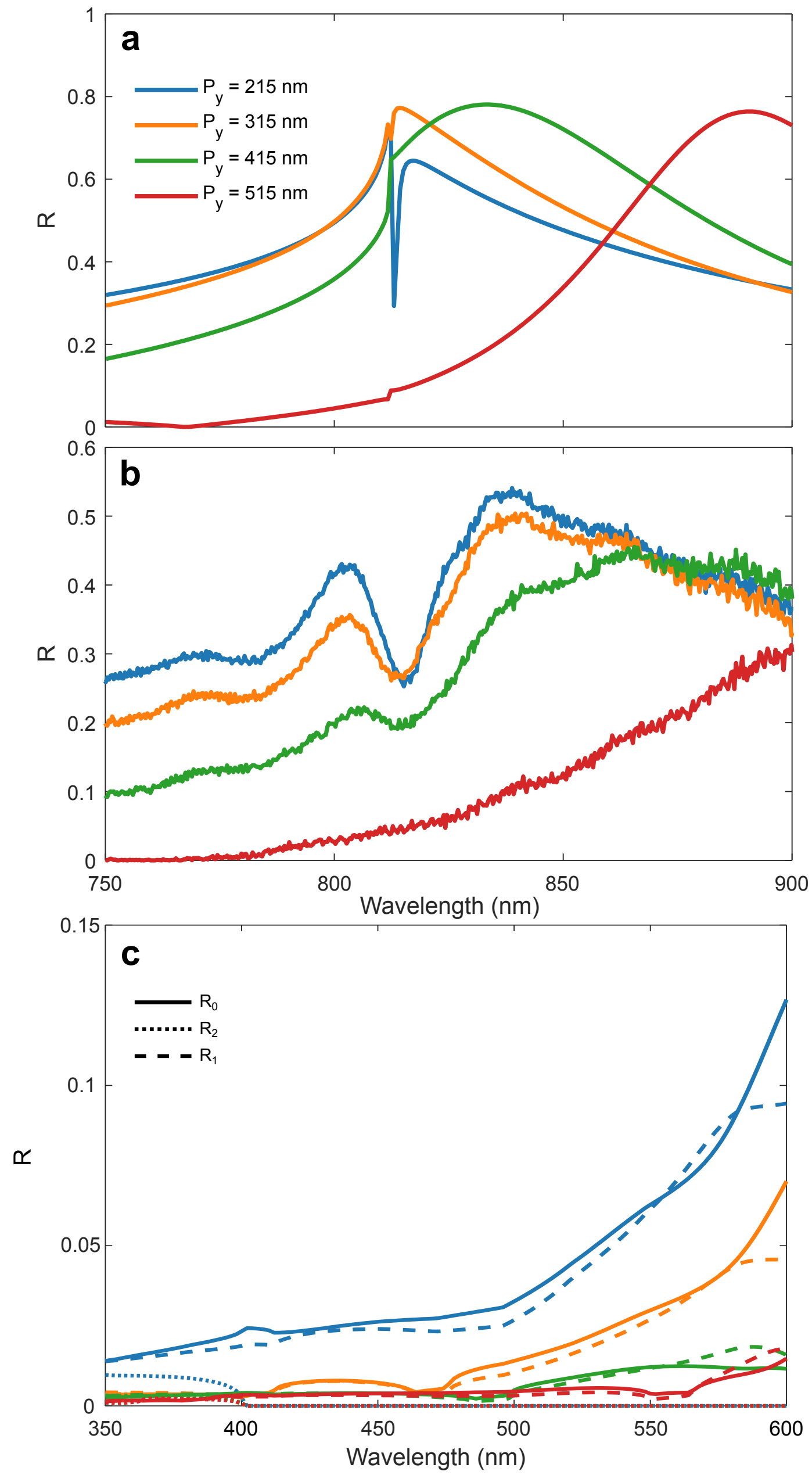

FIG. S2. **a** Simulated and **b** experimental linear reflection spectra of the samples with varying P_y parameters (profiles of the pseudocolor plots in Figure 1b and 3b, plotted on a line plot for clarity). **c** Simulated linear refection spectra at the second-harmonic frequency (including diffracted orders, denoted with dashed and dotted lines). The same color coding to is used to denote P_y values as in Figure 4.

S3. COMPONENT-WISE MULTIPOLE DECOMPOSITION

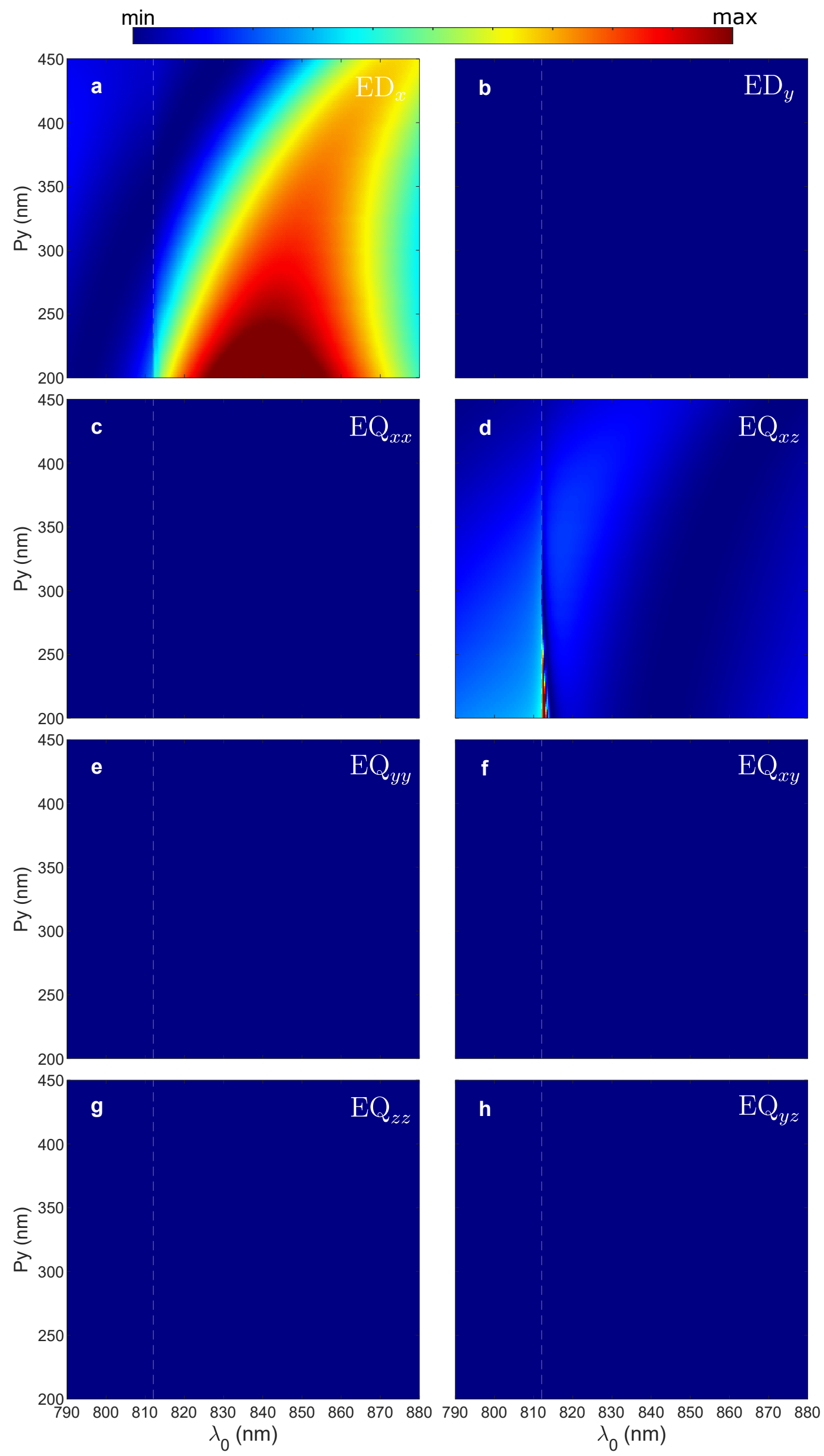

FIG. S3. Amplitudes of the electric dipole and quadrupole moment components: **a** ED_x, **b** ED_y, **c** EQ_{xx}, **d** EQ_{xz}, **e** EQ_{yy}, **f** EQ_{xy}, **g** EQ_{zz}, **h** EQ_{yz}. Only ED_x and EQ_{xz} moments are contributing to the metasurface response, the rest are practically negligible. All plots are normalized to the same maximum. Note: the scale and units are different as compared with Fig. 2, where multipole intensities are plotted.

S4. NONLINEAR MICROSCOPY SETUP

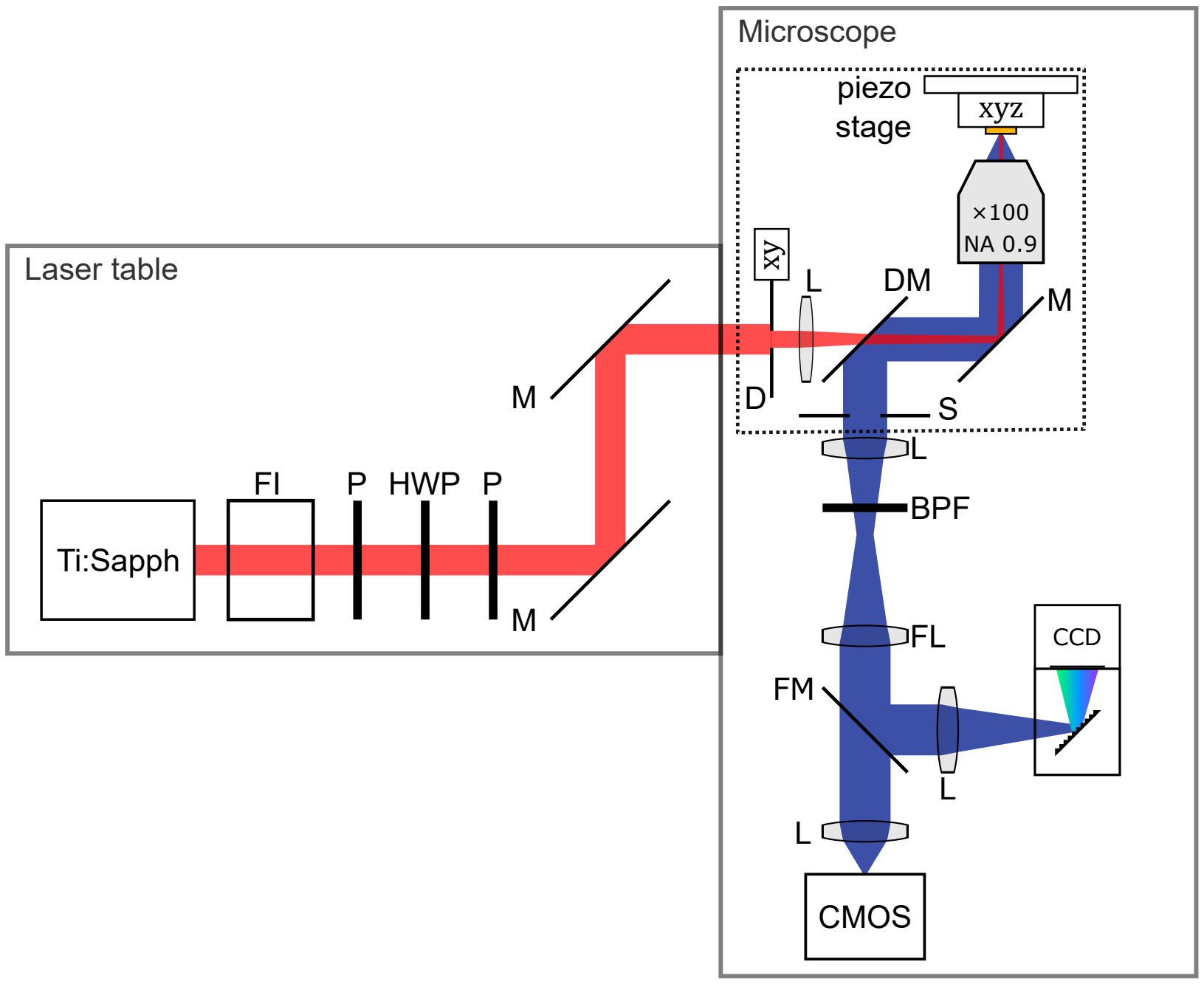

FIG. S4. Schematics of the custom-made scanning optical microscope used for nonlinear measurements. Red color is used for the fundamental beam; blue color is used for the nonlinear beam. The following components are indicated: Faraday isolator (FI); polarizer (P); half-wave plate (HWP); mirror (M); iris diaphragm (D); slit (S); lens (L); flip lens (FL); flip mirror (FP); band pass filter (BPF); dichroic mirror (DM); spectrograph with a charge-coupled device camera (CCD); complementary metal-oxide semiconductor camera (CMOS). Quasi-plane-wave like illumination is achieved by closing the iris diaphragm D and displacing it in the plane of incidence, filling only a portion of the objective's back focal plane. Further details on the used equipment are provided in the Methods section of the main text.

S5. POWER AND SPECTRAL DEPENDENCE OF THE NONLINEAR SIGNAL

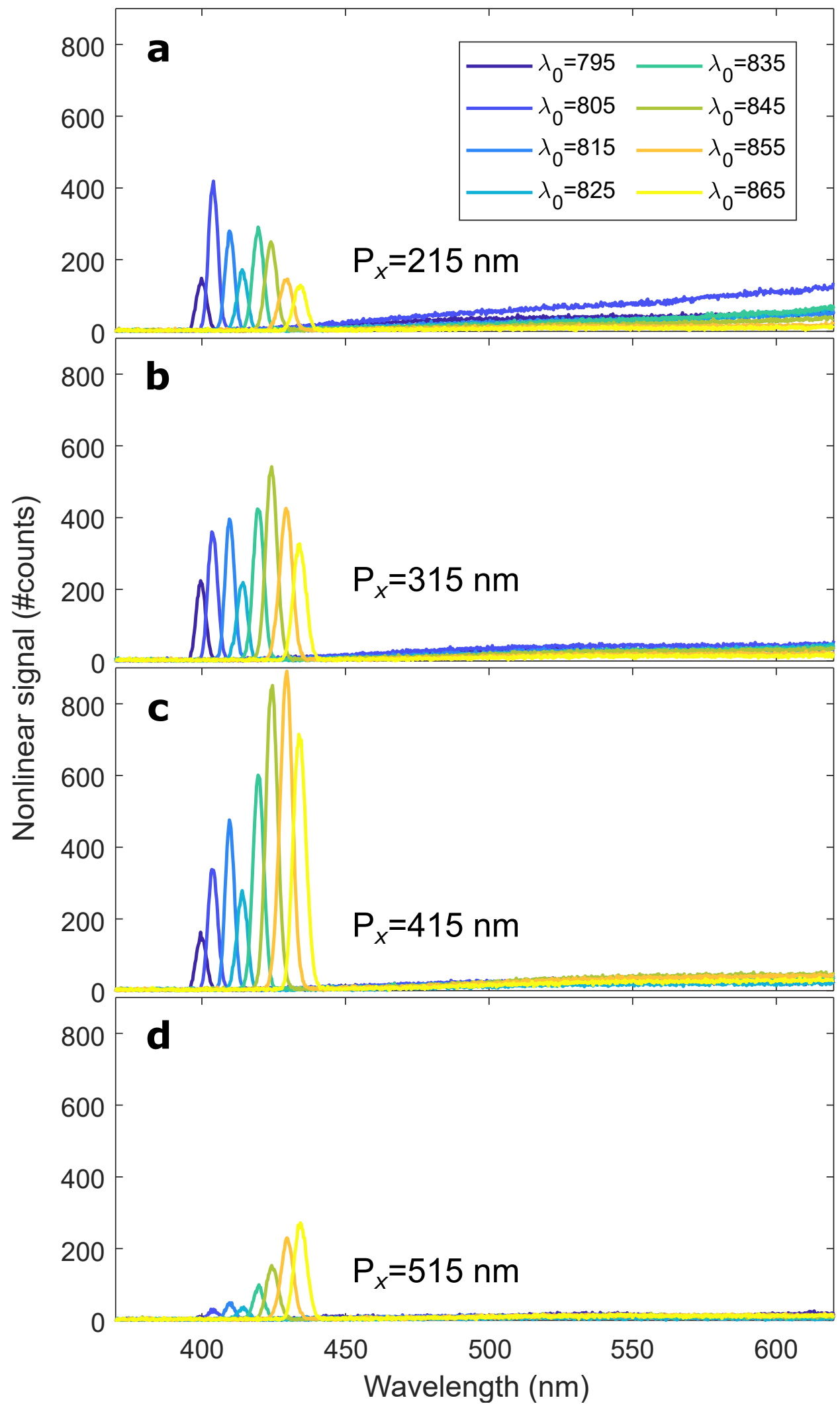

FIG. S5. Measured raw nonlinear spectra of the samples with varying $P_x = 215\,\mathrm{nm}$ (**a**), $315\,\mathrm{nm}$ (**b**), $415\,\mathrm{nm}$ (**c**), $515\,\mathrm{nm}$ (**d**).

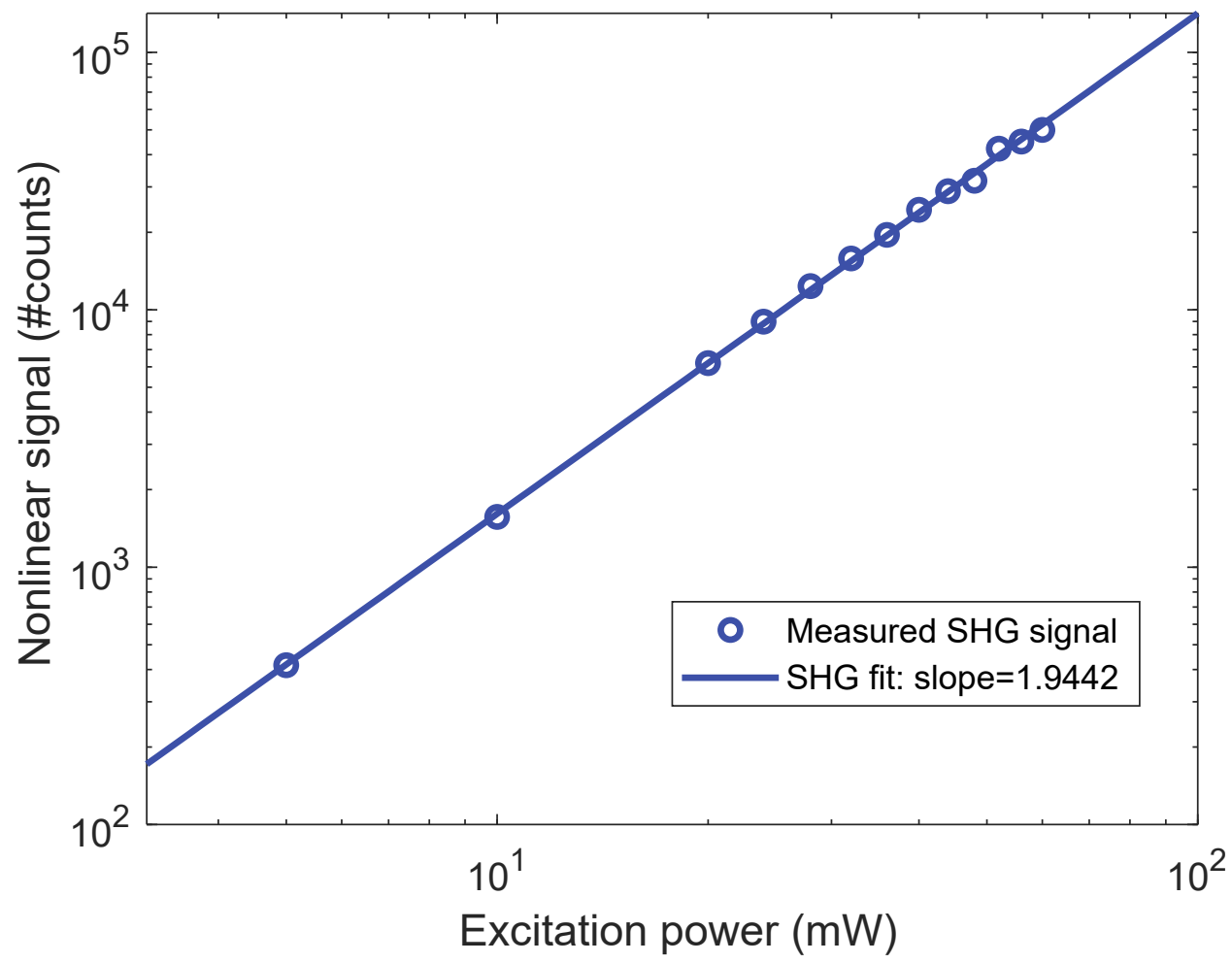

FIG. S6. Power dependence of the SHG signal. A regression fit of the $\log_{10}$ of the signal gives the value 1.94, which implies that the intensity of SHG scales nearly quadratically with the excitation power. The measurement was performed at 820 nm excitation wavelength (corresponding to 410 nm SH wavelength) with $P_y = 215\,\mathrm{nm}$ sample

S6. CHARACTERIZATION OF THE EXCITATION AND SHG SPOT

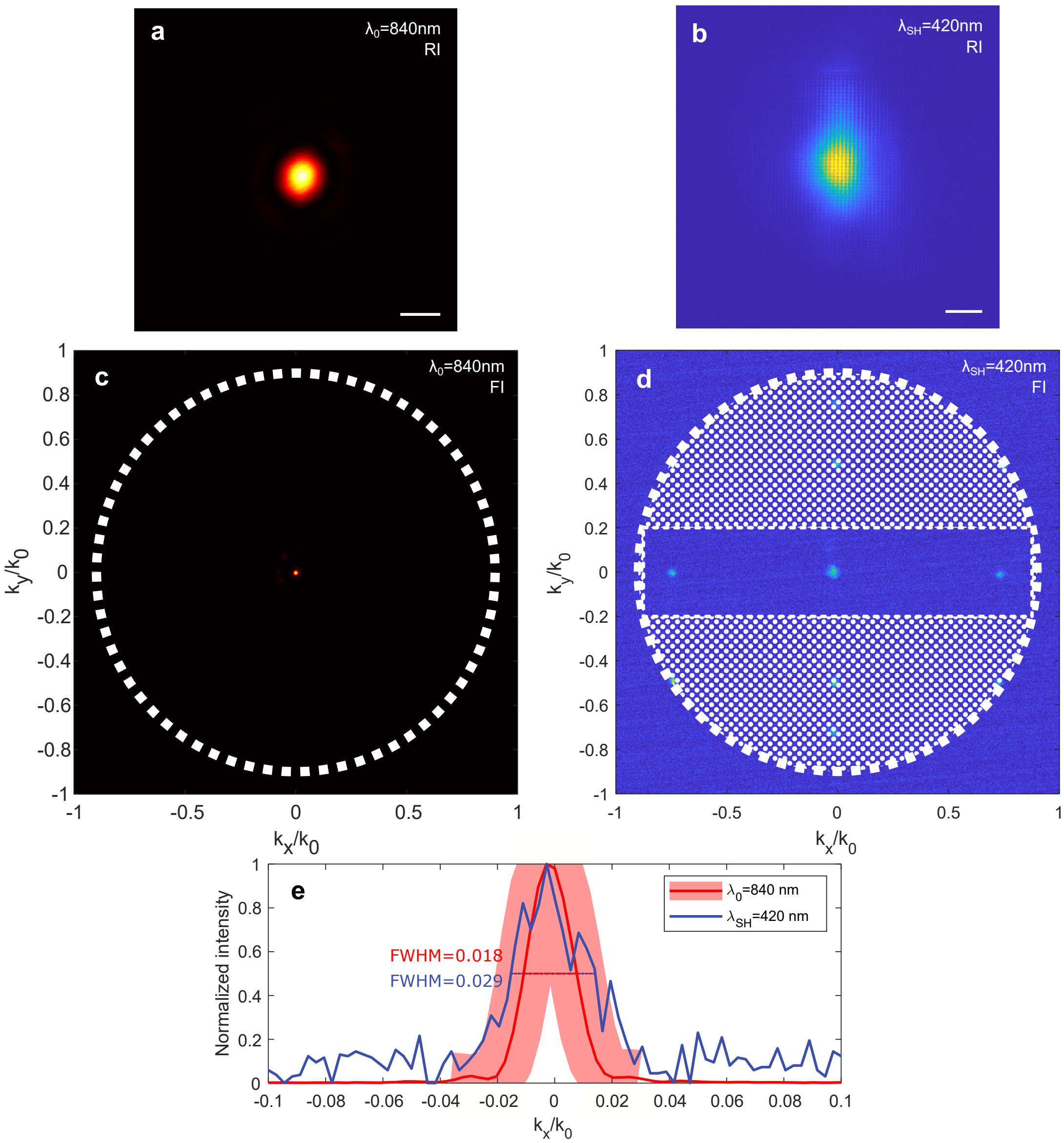

FIG. S7. **a**, **b** Real space images of the excitation ($\lambda_0 = 840\,\text{nm}$, **a**) and SHG ($\lambda_0 = 420\,\text{nm}$, **b**) from a metasurface with $P_x = 415\,\text{nm}$; scale bars: $5\,\mu\text{m}$. **c**, **d** Fourier images of the excitation (**c**) and SHG (**d**); only the signal in unshaded area was used in spectral measurements in Fig. S5. **e** Intensity profiles extracted from the Fourier images in **c** and **d**. In terms of the normalized wave-number (k_x/k_0), the calculated full width at half maximum (FWHM) of the fundamental and SHG spots are 0.018 and 0.028, which correspond to 0.51° and 0.83° half-angles respectively. Shaded (pink) area indicates approximate uncertainty of the center of the excitation spot along the k_x/k_0 axis, which is comparable with the FWHM of the signal.
